# The Spin1 interactor, Spindoc, is dispensable for meiotic division, but essential for haploid spermatid development in mice

**DOI:** 10.1186/s12958-021-00828-8

**Published:** 2021-09-15

**Authors:** Xue Jiang, Xiaoli Zhu, Yu Cheng, Muhammad Azhar, Xuemei Xing, Wenqing Li, Yuzhu Cao, Qinghua Shi, Jianqiang Bao

**Affiliations:** Division of Life Sciences and Medicine, The First Affiliated Hospital of USTC, University of Science and Technology of China (USTC), Hefei, Anhui 230001 People’s Republic of China

**Keywords:** Spindoc, c11orf84, spermatogenesis, germline, spermatids, meiosis, spermiogenesis

## Abstract

In mammals, germline development undergoes dramatic morphological and molecular changes and is epigenetically subject to intricate yet exquisite regulation. Which epigenetic players and how they participate in the germline developmental process are not fully characterized. Spin1 is a multifunctional epigenetic protein reader that has been shown to recognize H3 “K4me3-R8me2a” histone marks, and more recently the non-canonical bivalent H3 “K4me3-K9me3/2” marks as well. As a robust Spin1-interacting cofactor, Spindoc has been identified to enhance the binding of Spin1 to its substrate histone marks, thereby modulating the downstream signaling; However, the physiological role of Spindoc in germline development is unknown. We generated two *Spindoc* knockout mouse models through CRISPR/Cas9 strategy, which revealed that *Spindoc* is specifically required for haploid spermatid development, but not essential for meiotic divisions in spermatocytes. This study unveiled a new epigenetic player that participates in haploid germline development.

## Introduction

In mammals, the production of functionally competent sperm is a lengthy and complex biological process, which is in general divided into three successive stages – the proliferation and differentiation of spermatogonia derived from neonatal gonocytes, one time of DNA replication followed by two times of cell divisions, termed meiosis, and subsequent haploid germline development, also named spermiogenesis [1]. To achieve this end, this whole developmental process necessitates the expression of abundant germline-specific or -predominant genes in testis, including those genes encoding the structural components of acrosomes and tails of sperm cells [2]. On the other hand, the germline undergoes a drastic and sophisticated process of epigenetic programming, such as the dynamic chromatin remodeling and histone modifications, which requires a rich set of testis-preferential epigenetic modifiers [1]. These enable the deposition (writer), recognition (reader), and removal (eraser) of specific histone post-translational modifications (PTMs) primarily residing in N-terminal histone tails. These combinatorial modifications render the transition of local chromatin between a closed, transcriptionally inert state and an open, transcription-permissive state, thereby facilitating the fine-tuning of gene expression in response to developmental cues. Spindlin1 (Spin1) is a transcriptional coactivator that comprises three Spin/Ssty motifs, which are individually folded resembling a Tudor-like β-barrel conformation [3–5]. Early biochemical studies revealed that Spin1 is a multifunctional histone reader with the second Tudor module recognizing the H3K4me3/H4K20me3 while the first one binding H3R8me2a, which stimulates gene expression involved in ribosomal DNA (rDNA) transcription, Wnt/β-catenin signaling and MAZ (Myc-associated zinc finger protein) target gene activation [6, 7]. More recently, structural studies have shown that Spin1 recognizes a bivalent histone methylation signature, H3” K4me3-K9me3/2″, with a four-fold higher binding affinity, than H3 “K4me3-R8me2a” signature [8, 9]. H3K4me3 was traditionally regarded as an active histone mark for transcriptional activation, whereas H3K9me3/2 function as canonical repressive histone marks; However, they are not mutually exclusive, but indeed coexist in coding genomic regions of rDNA with active transcription [10]. In agreement with this, the histone H3K9 demethylase KDM7B (PHF8) harbors PHD and JmjC domains, and has been shown to activate rDNA transcription in nucleoli, through the PHD module recognizing H3K4me3 and JmjC domain promoting the H3K9me3/2 demethylation *in cis* [11]. Intriguingly, recent studies implicated that both H3K4me3 and H3K9me3 are present in haploid genome and participate in transgenerational epigenetic inheritance in mice and *C. elegans* [12, 13].

Spin1 was initially identified as a maternal protein highly expressed in oocytes and embryos at the 2-cell stage in mice. Subsequent studies have shown that Spin1 is dispensable for folliculogenesis, but is required for meiotic division in female mice [14, 15]. In porcine oocytes, Spin1 is localized in both the cytosol and the nucleus, and maintains the MII-arrested state [16]. While the functional mechanism of Spin1 remains not fully characterized, recent studies identified that the in vivo Spin1-interacting protein, C11orf84, also termed Spindoc, modulates the transcriptional co-activity of Spin1 through its interaction with the third Tudor domain of Spin1, raising a possibility that *Spindoc* might play an important role in germline development [9, 17].

Here we generated *Spindoc* knockout mouse models via CRISPR/CAS9, and reported that mouse Spindoc is not required for meiotic divisions in spermatocytes, but is essential for the haploid spermatid development after meiosis. *Spindoc*-deficient males displayed subfertility owing to the decreased sperm number and abnormal sperm morphology. This study added a new epigenetic player that exerts pivotal roles in germline development.

## Results

### Spindoc is predominantly expressed in testis

**Fig. 1:**
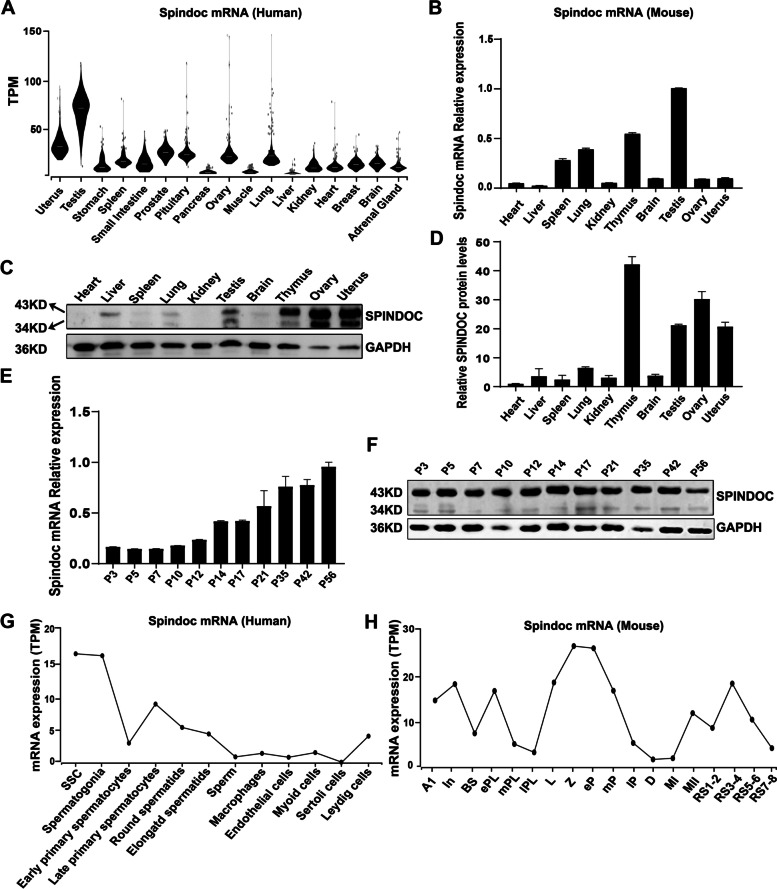
Spindoc is predominantly expressed in testis. **(A)** Violin plots showing the mRNA expression pattern of Spindoc in multiple human organs in the GTEx database (TPM, Transcripts Per Million). **(B)** RT-qPCR analyses of Spindoc mRNA levels across ten organs from adult wild-type (WT) mice. Data are presented as mean ± SEM, *n* = 3. (**C**) Western blot showing the expression levels of Spindoc protein among ten organs of WT adult mice. GAPDH served as a loading control. (**D**) Densitometric quantification of Spindoc protein levels as in (**C**). Data are presented as mean ± SEM, n = 3. (**E**) RT-qPCR analyses of Spindoc mRNA levels in developing testes. Testes at postnatal Day 3 (P3), P5, P7, P10, P12, P14, P17, P21, P35, P42 and P56 were analyzed. Data are presented as mean ± SEM, *n* = 3. (**F**) Western blot illustrating the Spindoc protein levels in developing testes. Testes at postnatal Day 3 (P3), P5, P7, P10, P12, P14, P17, P21, P35, P42 and P56 were analyzed. GAPDH served as a loading control. (**G**) Dynamic expression pattern of Spindoc mRNA from single cell RNA-seq analyses in adult human testes [18]. SSC, Spermatogonial Stem cells. (**H**) Dynamic expression levels of Spindoc mRNA from single cell RNA-seq analyses in RA-synchronized testicular cells [19]. A1, type A1 spermatogonia; In, intermediate spermatogonia; BS, S phase type B spermatogonia; ePL, early preleptotene; mPL, middle preleptotene; lPL, Late preleptotene; L, leptotene; Z, zygotene; eP, early pachytene; mP, middle pachytene; lP, late pachytene; D, diplotene; MI, metaphase I; MII, metaphase II; RS1–2, steps 1–2 spermatids; RS3–4, steps 3–4 spermatids; RS5–6, steps 5–6 spermatids; RS7–8, steps 7–8 spermatids

To study the functional roles of Spindoc in germline development, we firstly examined the multi-tissue expression patterns of Spindoc in humans and mice. GTEx database has revealed the highest levels of *Spindoc* transcript in the testis, as compared to that in other somatic tissues, in humans (Fig. 1 A). Consistently, the quantitative PCR (qPCR) assays showed predominant expression of *Spindoc* mRNA transcript in testis, as compared in other somatic organs, in mice (Fig. 1 B). At the protein level, the expression levels of Spindoc slightly differed from that of its mRNA abundance among different tissues, with testis being one of the strongest expression organs in mice, suggesting protein translation of Spindoc is subject to post-transcriptional regulation (Fig. 1 C,D). In mammals, germline development is strictly time-defined, with different stages of germ cells occurring at specific time points. Thus, we next investigated how Spindoc is expressed during postnatal germline development. As shown in Fig. 1 E, while the Spindoc mRNA displayed an increased expression trend during spermatogenesis, its protein expression sustained a relatively high level throughout postnatal testicular development (Fig. 1 F), suggesting that the mRNA and protein of Spindoc are present from spermatogonia to post-meiotic spermatids. Further single cell RNA-seq analyses validated that Spindoc displays a highly dynamic mRNA expression pattern at various developmental stages of germ cells, with higher mRNA levels detected in early spermatogonia, late spermatocytes and haploid spermatids in both humans (Fig. 1 G) and mice (Fig. 1 H) [18, 19]. Together, these evidences suggest that Spindoc might play important roles in germline development.

### Generation of *Spindoc* knockout mouse models

**Fig. 2:**
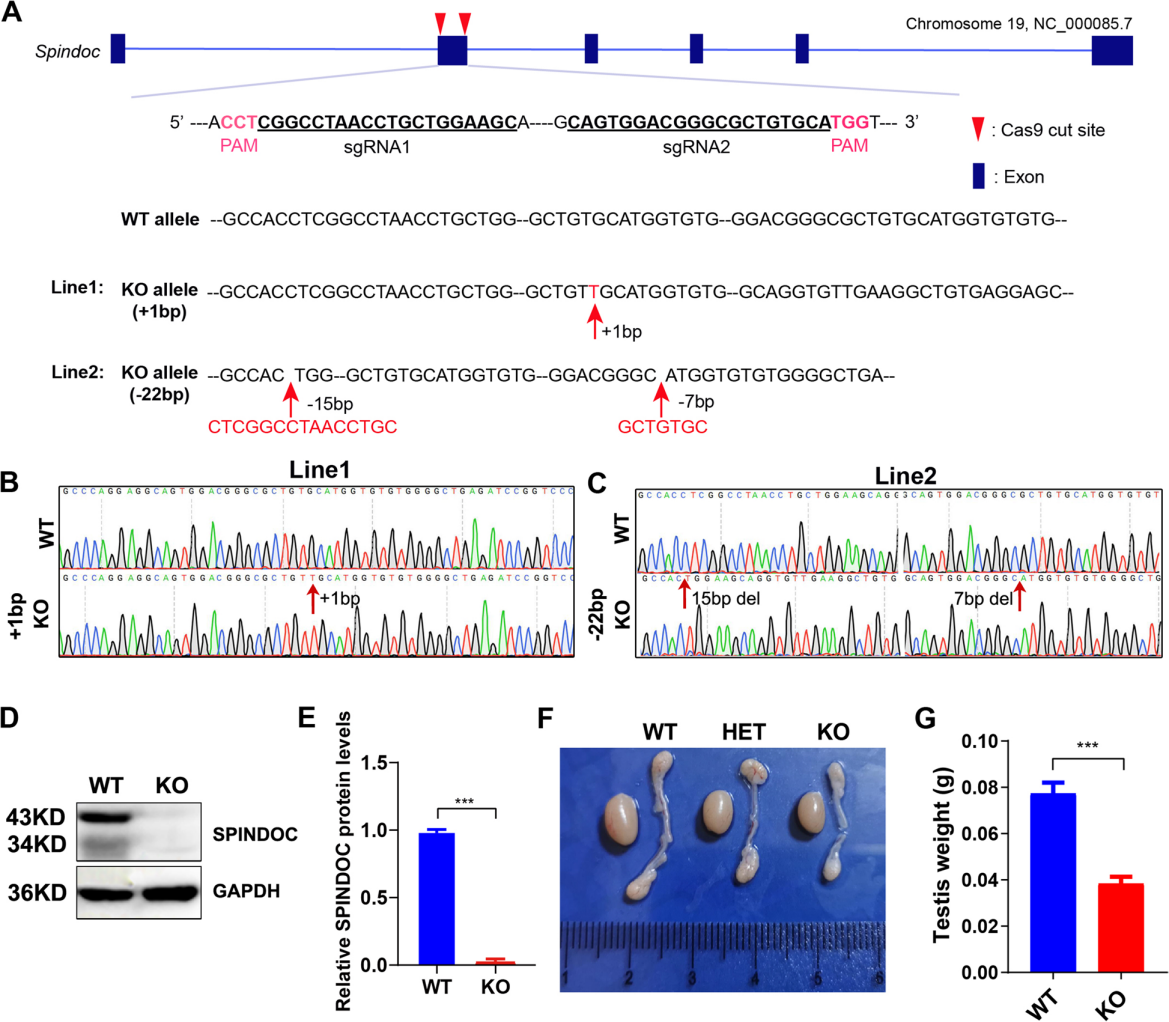
Generation of *Spindoc* knockout mouse models. (**A**) Schematic diagram shows the targeting strategy for generating *Spindoc* knockout (KO) mice using the CRISPR/Cas9 system. Blue boxes represent exons of the *Spindoc* gene on mouse chromosome 19. The genomic sequence targeted by a pair of sgRNAs are underlined with PAM sequences being highlighted in purple color. The red arrowheads point to the cut sites around exon 2 of *Spindoc* gene. Two F0 founder lines were generated - Line 1 carried 1 nt (T) insertion, while Line 2 carried a combination of 22 bp deletions as indicated; (**B, C**) Sanger sequencing of genomic DNA from mouse tail clips showing the frameshift variant (+ 1 nt) of KO mice (Line 1) (B), and the other frameshift variant (− 22 nt) (Line 2) (C); (**D**) Representative western blot analyses validated the Spindoc protein levels in WT and KO adult testes. GAPDH was used as a loading control. (**E**) Quantitative analysis of Spindoc protein levels in WT and KO adult testes. Data are presented as mean ± SEM, *n* = 3. *P* < 0.001 by *student t-test*. (**F**) Representative morphology of testes and epididymis from WT, Heterozygote (HET) and KO mice. The testis of KO was smaller than that of the WT; the epididymis of KO was more transparent than that of the WT. (**G**) Histogram showing the testis weights in WT and KO adult mice. Data are presented as mean ± SEM, n = 3. *p* < 0.001 by *student t-test*

To investigate the in vivo function of Spindoc, we next chose to generate a mouse model deficient in *Spindoc* through the CRISPR/CAS9 technology. We designed a pair of sgRNAs, which targeted the exon2 of *Spindoc* gene (Fig. 2 A). Cas9 mRNA and sgRNAs were microinjected into fertilized zygotes. The two-cell embryos were subsequently transferred into surrogate pregnant mothers. After birth, Sanger sequencing validated that we obtained two different founder lines: one line carrying a single nucleotide (T) insertion in exon2 (Fig. 2 B) (Hereafter called Line 1), and the other harboring a combination of 22 bp deletion of exon2 (Fig. 2 C) (Line 2). Both types of mutations resulted in the presence of premature STOP codon, thus leading to the activation of nonsense-mediated mRNA decay (NMD) pathway. The F0 founder mice were crossed with WT females to achieve the heterozygous offspring, which were further inter-crossed to gain *Spindoc-null* (KO) pups. Western blot analyses confirmed that we successfully generated *Spindoc-null* mice (Fig. 2 D,E). Morphological examination demonstrated that *Spindoc-null* testes exhibited reduced weight as compared to that in WT littermates, indicative of impaired germline development postnatally (Fig. 2 F,G). Given that both Line 1 and Line 2 KO males exhibited the similar phenotype, we henceforth focused on Line1-derived offspring in our subsequent studies.

### *Spindoc* KO caused impaired sperm production leading to male subfertility

**Fig. 3:**
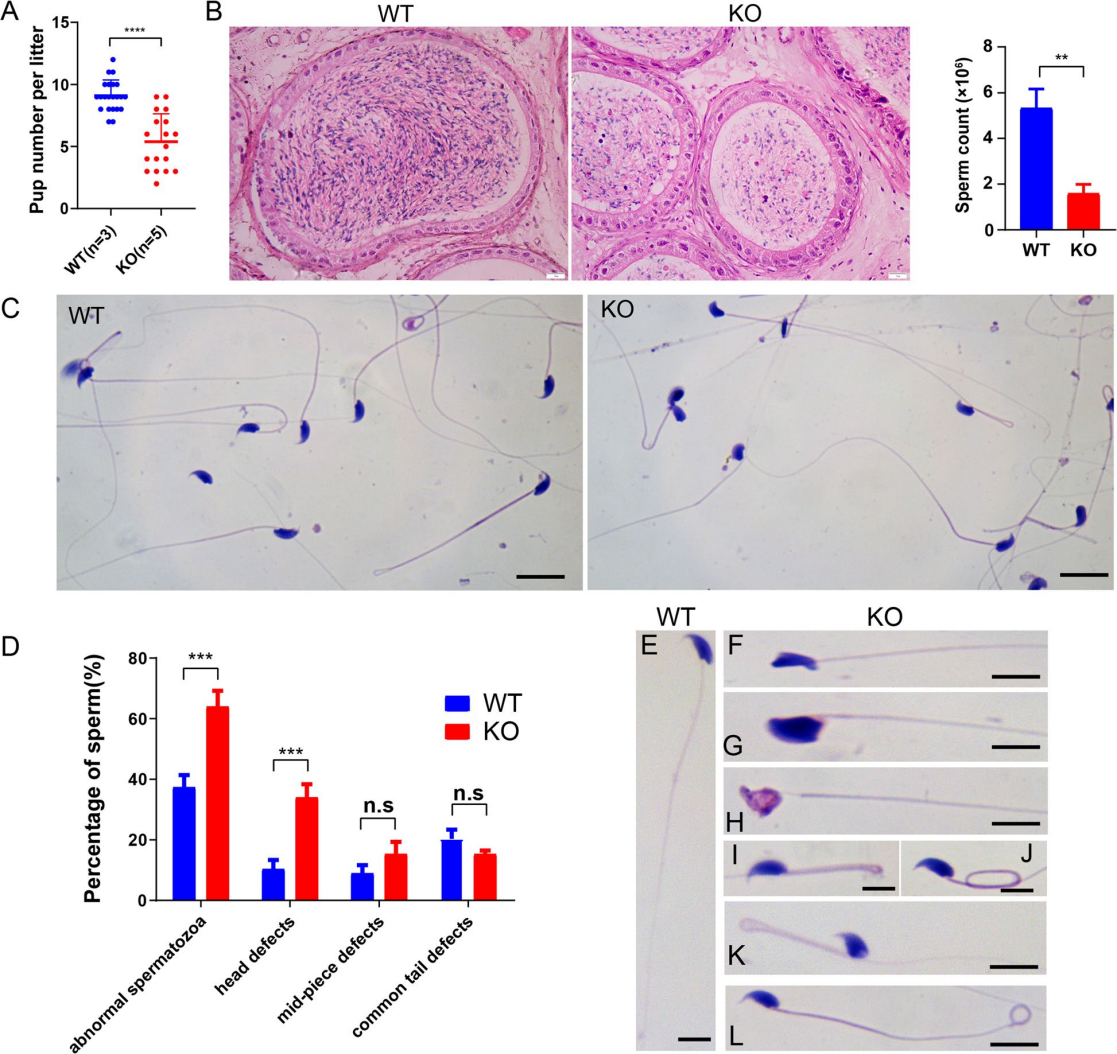
*Spindoc* KO caused impaired sperm production leading to male subfertility. (**A**) Number of pups per litter from male mice (> 8 weeks) naturally crossed with WT female mice (> 6 weeks) for 4 months. Data are presented as the mean ± SD, *n* = 5, *p* < 0.0001 by *student t-test*. (**B**) Histological analysis of the cauda epididymis from the WT and KO mice. The average number of sperm released from the cauda in KO mice was less than that in WT mice. Scale bar = 50 μm. The histogram showed the number of sperm retrieved from one cauda epididymis of WT and KO mice. Data were presented as mean ± SEM, n = 3. *P* < 0.01 by *student t-test*. (**C**) Histological analysis showing the morphology of sperm from cauda epididymis in WT and KO mice. Scale bar, 10 μm. (**D**) The histogram showing the percentage of sperm with abnormal morphology in WT and KO mice at the age of 6 months. Data are presented as mean ± SEM, n = 3. P < 0.001 by *student t-test*. (**E**) HE staining of sperm with normal morphology in the WT mice. Sperm with head defects, including irregular shape (**F, G, H**), coiled mid-piece tail (**I**, **J, K**), and tail defect (**L**), were more frequently observed in the KO mice. Scale bar, 5 μm

To test the effect of *Spindoc* KO on male fertility, we crossed the adult *Spindoc-null* males with WT females. Over a 4-month breeding period, we observed reduced litter size in *Spindoc-null* males as compared to in WT males (Fig. 3A), indicating the male subfertility upon *Spindoc* KO. Therefore, we next performed the Hematoxylin & Eosin (HE) staining on the cauda epididymis from the WT and *Spindoc-null* mice. In contrast to WT cauda as shown in Fig. 3B, the KO cauda was filled with a smaller number of condensed sperm. Detailed counting indicated that the total sperm number in *Spindoc-null* cauda is reduced to one third of that in WT cauda (Fig. 3B). Furthermore, careful examination of the sperm morphology by HE staining showed that *Spindoc-null* mice produced higher numbers of sperm with defects in the mid-piece and head condensation, suggesting defective haploid spermatid development (Fig. 3 C,D,E), suggesting that *Spindoc* KO possibly caused a developmental defect in the haploid spermatids.

### Intact meiotic divisions in spermatocytes in *Spindoc-null* mice

**Fig. 4:**
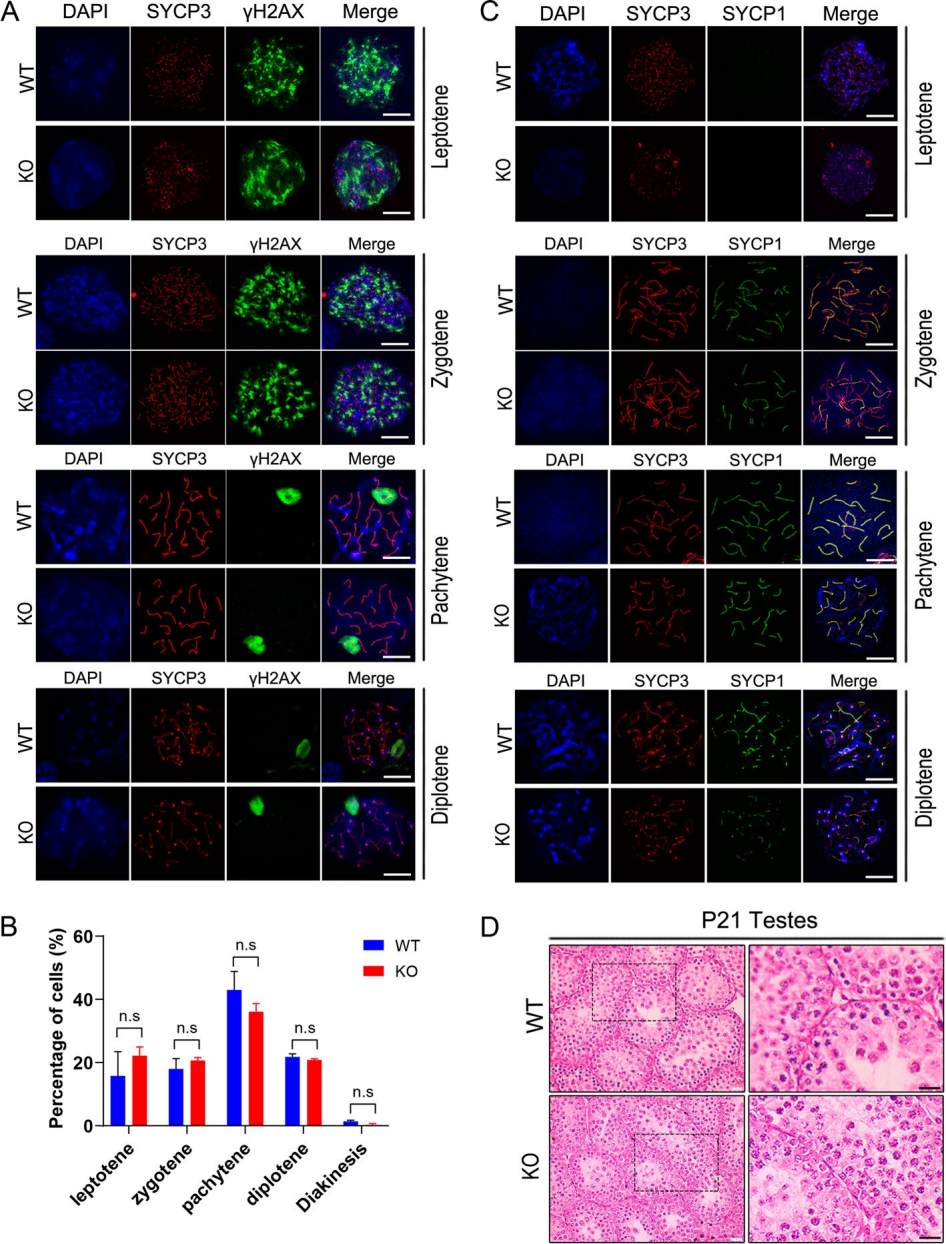
Meiotic divisions appeared to be normal in spermatocytes from *Spindoc-null* mice. (**A**) Immunofluorescence staining of SYCP3 (red) and γH2AX (green) on chromosome spreads of spermatocytes from the testes of WT and KO males at P21. DAPI indicates the nucleus. Scale bar, 10 μm. (**B**) Quantitative counting of the proportion of meiotic stages in WT and KO spermatocytes. Distinct meiotic stages (leptotene, zygotene, pachytene, diplotene and diakinesis) were identified by the characteristic patterns as shown in (A). For each genotype, three mice were analyzed. *p*-values were calculated by *Student’s t-test*. Data are presented as the mean ± SD; n.s., not statistically significant. (**C**) Immunofluorescence staining of SYCP3 (red) and SYCP1 (green) on chromosome spreads of spermatocytes from the testes of WT and KO males at P21. DAPI indicates the nucleus. Scale bar, 10 μm. (**D**) HE staining of the testes from the WT and KO mice at the age of 21 days. Areas with dash boxes were displayed with higher magnification on the right panel. Scale bar, 20 μm

Given that Spin1 has been previously shown to drive the meiotic resumption in oocytes [15, 16], we next determined to test if meiosis was impaired in *Spindoc-null* spermatocytes. To this end, we performed the immunofluorescent staining using SYCP3 and γH2AX on chromosome spreads prepared from postnatal day 21 (P21) testes following a standard drying-down preparation protocol [20]. Surprisingly, the progression of meiotic prophase I in *Spindoc-null* spermatocytes appeared to be normal, resembling those observed in the WT spermatocytes without any discernable morphological abnormality (Fig. 4 A), which is not consistent with the reported function of Spin1 in meiotic division of oocytes previously [15]. The percentage of *Spindoc-null* spermatocytes at various stages was comparable to that in WT testis (Fig. 4 B). We further executed the double staining with SYCP1 and SYCP3, which labelled the central and lateral synaptonemal complex filaments, respectively. These further corroborated that the paring and synapsis between homologous chromosomes in *Spindoc-null* spermatocytes were indistinguishable from those in WT spermatocytes (Fig. 4 C). In accord with this finding, Hematoxylin and Eosin (HE) staining demonstrated the normal meiotic progression in *Spindoc-null* testes, as evidenced by the presence of round spermatids in the seminiferous tubules (Fig. 4 D) in P21 testes. Together, these data suggest that *Spindoc* is not necessary for meiotic divisions in spermatocytes in mice.

### Defective transition from round spermatids to elongated spermatids

**Fig. 5:**
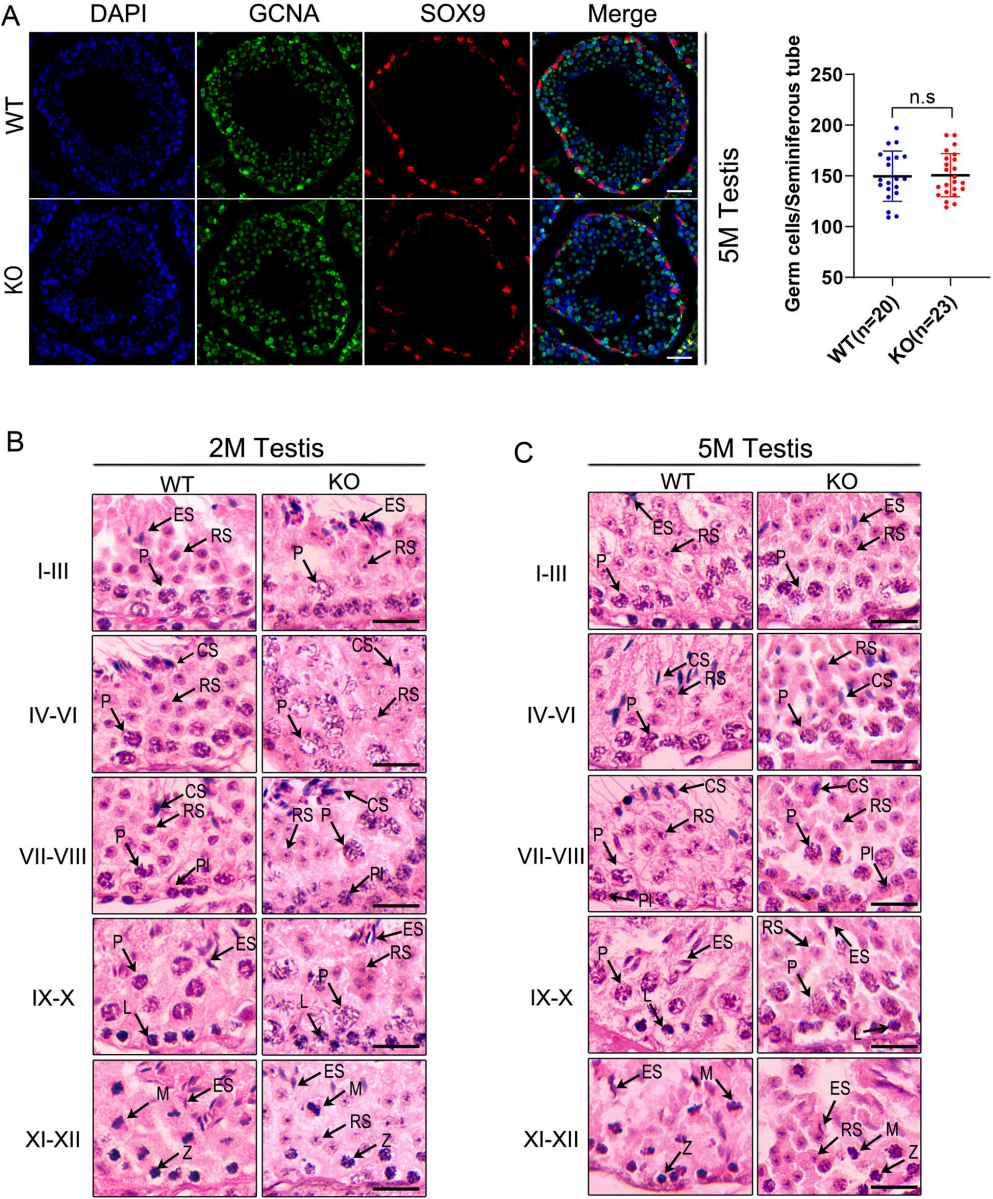
Defective elongation of round spermatids in the *Spindoc-null* mice. (**A**) Immunofluorescence staining of testicular sections from WT and KO mice at 5 months of age demonstrating no significant loss of germ cells in the KO mice. Scale bar, 20 μm. Quantification of germ cells was performed in each seminiferous tubule of WT(*n* = 20) and KO(*n* = 23) at 5 months of age (right panel). Data were presented as the mean ± SD, *p* > 0.1. (**B**) HE staining of seminiferous tubules from the WT and KO mice at the age of 2 months, indicating interrupted transition from round spermatids to elongated spermatids. Between stages IX- XII, round spermatid-like (RSL) cells were present in the KO testes; Pl, pre-leptotene; L, leptotene; Z, zygotene; P, pachytene; RS, round spermatids; RSL, round spermatid-like; CS, condensed spermatids; ES, elongated spermatids; M, metaphase. Scale bar, 20 μm. (**C**) HE staining of seminiferous tubules from the WT and KO mice at the age of 5 months, indicating defective elongation of round spermatids (stages IX-XII) in the KO mice. Scale bar, 20 μm

As described above, *Spindoc-null* spermatocytes appeared to progress normally during meiosis as observed in WT testes; However, the declined sperm count and aberrant sperm morphology suggested that there must be post-meiotic defects occurring during the haploid spermatid development. To test this hypothesis, we first examined the cell number ratio of the germ cells to Sertoli cells through the co-staining with respective markers, GCNA and SOX9, in 5-month testes. Not surprisingly, we didn’t observe noticeable changes in the ratio of germ cells to Sertoli cells (Fig. 5A). In mice, spermatogenesis takes place in successive waves along the epithelia of seminiferous tubules [2]. In any cross-section of the tubules, there is a total of 12 stages (I ~ XII stage) consisting of germ cells at various developmental steps lining up from the basal membrane to the lumen of the tubules [21]. Therefore, the comparison of the staging on the basis of the cellular morphology and associations between WT and KO testes was commonly adopted to pinpoint the specific developmental defects during spermatogenesis. We thus subsequently performed the HE staining on the paraffin-embedded testicular sections, and compared the spermatogenic stages between WT and KO testes from 2-month- and 5-month-old mice side by side (Fig. 5 B,C). At 2 months, there appeared to be similar number of round spermatids (RS) between Stages I ~ VIII, albeit with more aberrant elongated spermatids (ES) and condensed spermatozoa (CS) in KO seminiferous tubules as compared to in WT testes (Fig. 5 B). Intriguingly, when spermatogenesis moved progress beyond stage VIII, the elongating or elongated spermatids were present between Stage IX ~ XII in WT testes, whereas abundant round spermatids-like (RSL) germ cells were observed in KO testes (Fig. 5 B). This finding indicated a developmental arrest occurring during the transition from round spermatids to elongated spermatids upon *Spindoc* KO. The similar developmental arrest was further recapitulated in testes from 5-month-old mice (Fig. 5 C). Taken together, these evidences suggest that the development of haploid spermatids was disrupted in the absence of *Spindoc* in male mice.

## Discussion

Chromatin modifiers, including the epigenetic writers, readers, and erasers for specific histone post-translational modifications (PTMs), have been discovered to play significant roles during germline development. Spin1 was originally identified as a highly abundant maternal protein in mouse oocytes, and was shown to resume meiotic divisions of fully grown oocytes at the GV stage in the juvenile female mice [15]. It is almost solely comprised of three tandem Tudor-like domains, of which the first two Tudor domains engage in the recognition of cis-tail H3 “K4me3-R8me2a” histone marks. Until recently, the third Tudor module was identified to strongly bind a cofactor, namely Spindoc, in vivo [17, 22]. However, how Spin1 and its cofactor, Spindoc, function during male germline development is largely unknown. In somatic cells, it has been shown that Spin1 is localized to the nucleoli, where it is highly enriched in the rDNA repeats with active transcription, thereby promoting the expression of rRNA genes [6]. In addition, ectopic overexpression of Spin1 led to the transformation of NIH3T3 cells showing disruption of the cell cycles and chromosomal instability [23]. Furthermore, Spin1 expression was elevated in some types of cancers, including the seminoma [24]. Our comprehensive examinations indicate that Spindoc is preferentially expressed in testes as compared to in other somatic tissues. Interestingly, the mRNA expression levels of Spindoc exhibit a highly dynamic pattern ranging from spermatogonia to late stage of spermatids (Fig. 1). In line with the Spindoc expression pattern, the Spin1 mRNAs were also highly detected in the spermatogonia as well as in the spermatocytes (data not shown). These evidences suggest that Spin1/Spindoc complex might coordinate and execute pivotal roles in germ cells, in particular at the stage of meiosis, as seen in the female oocytes [14]. However, to our surprise, in the *Spindoc* KO mouse models, we observed only the post-meiotic defects during spermiogenesis but not the meiotic anomaly. Haploid spermatids were arrested during the transition from round spermatids to elongating and elongated spermatids. Additionally, the condensed spermatozoa that “escaped” the developmental arrest exhibited aberrant morphological defects, including the misshapen heads and abnormal midpiece of the sperm tails, reminiscent of the disrupted nuclear condensation of the spermatozoa head in the absence of *Spindoc*. Taken together, our study revealed an epigenetic factor, Spindoc, is essential for post-meiotic haploid germ cell development in mammals.

## Materials and methods

### Generation of the *Spindoc* knockout mouse model

The *Spindoc* knockout mouse model with the premature STOP codon was generated via the CRISPR/Cas9 technology through zygotic microinjection of the Cas9 mRNA and sgRNAs mixture. One pair of single-guide RNAs (sgRNAs) were designed against exon 2 of *Spindoc* (*NM_001033139.3*). Sanger sequencing was used to identify the genotypes of the offspring. Two founder mouse lines were validated by Sanger sequencing. One line carried one base pair (T) insertion of exon2, the other harboring combinatorial deletions as indicated (∆15 bp and ∆7 bp occurring at the sites targeted by the two sgRNAs, respectively); The sequences of the sgRNAs and the genotyping primers are listed in Table 1. All Mice were from the C57BL/6 J genetic background, and were bred in a specific pathogen-free (SPF) facility with a 12 h light/dark cycle and free access to food and water. All animal experiments have been approved by the Animal Care and Use Committee of University of science and technology in China.

**Table 1:** Primers used in this study

Gene name	Sequence(5′-3)	Application
Spindoc	ATGCTGGTTGGCTAGGATCT	Genotyping
	CTGGTCAGCTTGGATGTCTT	
Spindoc	GGTGGTGGCCGTGATCC	RT-qPCR
	CTGTTCCCCACCAGGAACTC	
Spindoc	CGGCCTAACCTGCTGGAAGC	sgRNA
	CAGTGGACGGGCGCTGTGCA	

### RNA extraction, reverse transcription and RT-qPCR

Total RNA was isolated from different tissues in mice with TRIzol Reagent following the manufacturer’s instructions as described previously [25]. Freshly collected or frozen tissues were homogenized in 1 ml TRIzol reagent per 50 mg tissue. The quantity and quality of RNA were determined by measurement using NanoPhotometer® N50 (Implen, Germany). The RNA samples with OD values of 260/280 ≥ 1.9 were selected for downstream analyses. To compare the *Spindoc* mRNA levels across tissues, equal amount of total RNAs was loaded to synthesize cDNAs using the RevertAid First Strand cDNA Synthesis kit (K1622, Thermo). Quantitative PCR (qPCR) was performed using ChamQ Universal SYBR qPCR Master Mix (Q711–02, Vazyme) with the Real-Time PCR machine (Roche). Primers for the qPCR were listed in Table 1.

### Western blot

Samples were freshly collected from different tissues in mice at different ages. Protein lysates were prepared in RIPA lysis buffer [100 mM Tris-HCl (PH7.4), 1% Triton X-100, 1% Sodium deoxycholate, 0.1% SDS, 0.15 M NaCl, supplemented with Protease inhibitor cocktail]. Protein concentrations were determined using the BCA protein assay kit. All protein samples were run in denatured 10 ~ 12% sodium dodecyl sulfate polyacrylamide (SDS-PAGE) gel, followed by the wet-transfer to PVDF membranes.

Membranes were blocked in 5% non-fat milk for 1 h at room temperature, and next were incubated with primary antibody overnight at 4 °C. After washing for three times in 1XPBS buffer, membranes were incubated with secondary antibody for 1 h at room temperature. The primary antibodies for immunoblotting included: rabbit anti-Spindoc (1:1000; PA5–65609; Invitrogen), rabbit anti-GAPDH (1:5000; 21,612; SAB).

### Sperm counting

Sperm were released from the cauda epididymis of adult mice by puncture using sharp forceps, and were incubated in HTF medium for 30 min at 37 °C. For KO mice, the cauda was slightly squeezed to push out sperm using a tweezer in order to minimize the retention of sperm inside the cauda owing to the declined motility of sperm in the KO mice. Sperm number was calculated using a hemacytometer. Smear slides of sperm were stained by standard Hematoxylin & Eosin to distinguish the morphological abnormality between WT and KO groups.

### Histology, immunofluorescent staining and chromosome spreads analyses

Hematoxylin & Eosin (HE) staining was performed following the standard procedure as described previously [25]. Testes were freshly collected and fixed in Bouin^**’**^s solution at room temperature overnight. Paraffin-embedded samples were cut into 5 μm slides. For immunofluorescence staining, testes were fixed in 4% paraformaldehyde (PFA) overnight at 4 °C using a rocker. Slides were dehydrated with 10 and 20% sucrose for 2 h respectively. For preparation of chromosome spreads, P21 mice were sacrificed, and testicular tubules were dispersed in hypotonic buffer [30 mM Tris-HCL (PH = 8.2), 50 mM sucrose, 17 mM trisodium citrate dihydrate, 5 mM EDTA (pH = 8.0), 1 mM dithiothreitol (DTT), and 1 mM phenylmethylsulfonyl fluoride (PMSF)] for ~ 30 min. Tubules were next transferred to 100 mM sucrose, and single cells were released into solution using sharp forceps. Single cells were spread and fixed in 1% PFA solution containing 0.15% Triton X-100 on slides. For antibody staining, slides were blocked with 5% BSA for 1 h at room temperature, and incubated with antibodies as follows: mouse anti-SYCP3 (1:1000; ab97672; abcam), rabbit anti-γH2AX (1:1000; ab2893; abcam), rabbit anti-SYCP1 (1:1000; ab15090; abcam), rat anti-GCNA1 (1:1000; ab82527; abcam), rabbit anti-SOX9 (1:500; A19710; ABclonal). Slides were imaged by Leica THUNDER Imager Live Cell with a K5 camera driven by the Leica Application Suite Software. Image processing was performed by ImageJ software. HE slides were imaged using a Mshot microscope (ML31) with a MSX2 camera.

### Statistical analysis

Statistical analysis was performed using *Student’s t test* unless stated elsewhere. The value of *p* < 0.05 was deemed significant statistically. Statistical data was processed by GraphPad Prism 6.

## Data Availability

Not applicable.

## Abbreviations

Spin1: Spindlin1
qPCR: quantitative PCR
NMD: nonsense-mediated mRNA decay
KO: knockout
